# Digital technology to support lifestyle and health behaviour changes in surgical patients: systematic review

**DOI:** 10.1093/bjsopen/zraa009

**Published:** 2020-12-28

**Authors:** A Robinson, A K Husband, R D Slight, S P Slight

**Affiliations:** 1 School of Pharmacy, Newcastle University, Newcastle upon Tyne, UK; 2 Newcastle upon Tyne Hospitals NHS Foundation Trust, Newcastle upon Tyne, UK

## Abstract

**Background:**

Digital technologies (such as smartphone applications, activity trackers, and e-learning platforms) have supported patients with long-term conditions to change their lifestyle health behaviours. The aim of this study was to examine the effectiveness of digital technologies in supporting patients undergoing elective surgery to change their health behaviours.

**Methods:**

A systematic review was conducted of articles reporting a digital intervention supporting behaviour change in adult patients who underwent elective bariatric, oncological or orthopaedic surgery. MEDLINE, Embase, CINAHL, PsycINFO, Web of Science, and Scopus were searched from inception to March 2019 for quantitative intervention studies with a specific focus on physical activity, dietary intake, and weight loss in patients before and after surgery (PROSPERO: CRD42019127972). The Joanna Briggs Institute critical appraisal checklist was used to assess study quality.

**Results:**

Of 3021 citations screened, 17 studies were included comprising 4923 surgical patients; these included experimental (pre–post design, feasibility studies, and RCTs) and observational studies. Three factors were identified as effective for supporting health behaviour change in elective surgical populations: digital technology delivery, implementation, and theoretical underpinning. Six of eight studies that referred to behaviour change theories observed significant improvements in health behaviour relating to reduced weight regain, and improved lifestyle choices for physical activity and diet. Meta-analysis was not possible because of heterogeneous outcome measures.

**Conclusion:**

Digital technologies may effectively support behavioural change in patients undergoing elective surgery.

## Resumen

**Antecedentes:**

Las tecnologías digitales (como las aplicaciones para teléfonos inteligentes, los rastreadores de actividad y las plataformas de aprendizaje electrónico) han ayudado a los pacientes con enfermedades crónicas a cambiar sus hábitos de salud y estilo de vida. El objetivo de este estudio fue examinar la efectividad de las tecnologías digitales para ayudar a los pacientes sometidos a cirugía electiva a cambiar sus comportamientos de salud.

**Métodos:**

Se realizó una revisión sistemática de los artículos que presentaban una intervención digital que apoyaba el cambio de comportamiento en pacientes adultos que se sometieron a cirugía bariátrica, oncológica u ortopédica electiva. Se realizaron búsquedas en las bases de datos Medline, Embase, CINAHL, PsycINFO, Web of Science y Scopus desde el inicio hasta marzo de 2019, para seleccionar estudios de intervención cuantitativos con un enfoque específico en la actividad física, la ingesta dietética y la pérdida de peso en pacientes antes y después de la cirugía (ver PROSPERO: CRD42019127972) Se utilizó la lista de verificación de evaluación crítica del Instituto Joanna Briggs para evaluar la calidad del estudio.

**Resultados:**

De 3.021 referencias examinadas, se seleccionaron 17 estudios con un total de 4.923 pacientes quirúrgicos, incluyendo estudios experimentales (diseño antes y después, estudios de viabilidad y ensayos aleatorizados y controlados) y estudios observacionales. Se identificaron tres factores como efectivos para apoyar el cambio de comportamiento de salud en poblaciones quirúrgicas electivas, incluida la entrega, la implementación y una base teórica de tecnología digital. Las tasas de retención de las intervenciones variaron del 55% al 100%. Seis de los ocho estudios que se refirieron a las teorías de cambio de comportamiento observaron mejoras significativas en el cambio de comportamiento de salud relacionado con la reducción de la recuperación de peso y mejores opciones de estilo de vida para la actividad física y la dieta. El metaanálisis no fue posible debido a las medidas de resultado heterogéneas.

**Conclusión:**

Las tecnologías digitales pueden apoyar eficazmente el cambio de comportamiento en pacientes sometidos a intervenciones quirúrgicas electivas.

## Introduction

Digital technologies are becoming an integral part of modern-day life. Recent reports from the UK Office of Communications and the Statista and the Office of Communications estimate that 78 per cent of adults own a smartphone, 90 per cent of people regularly access the internet in their home, 58 per cent own a tablet device, and 20 per cent use wearable technology, such as smart watches and fitness trackers^1^^,^^2^. A recent US-based review^3^ found that almost 60 per cent of American smartphone users have reported downloading and using fitness or health-related applications, more commonly termed apps. There has been a successful shift towards the integration of digital technologies in healthcare systems too. For clinicians, digital technologies can improve communication and information transfer between clinical teams and healthcare sectors^4^^,^^5^. For healthcare providers and organizations, digital technologies can assist in reducing the burden associated with working at increased capacity, and managing patients with increasing numbers of co-morbidities^6^^,^^7^. For patients, digital technologies can enhance education provision, improve communication with clinicians, and empower them to play an active role in their own care^4^^,^^8–10^. 

In a surgical context, recent evidence has linked better patient physical preparedness before surgery with improved outcomes and benefits after surgery^11–13^. More specifically, improvements in a patient’s dietary intake^14^, physical activity levels^15^, and smoking cessation^16^ have been linked to improved recovery, better tolerance of postoperative treatment, and prevention of related disease in the long term^8^^,^^17–19^. At present, variable amounts of support and education, however, are made available for patients undergoing elective surgery in order to motivate health behaviour changes. For instance, before weight loss surgery, patients are encouraged to change their diet and lose weight, but many feel unsupported^20–22^. Patients recovering from cancer surgery have previously reported poor lifestyle support and this has also been recognized by healthcare professionals^23^^,^^24^. To encourage changes to lifestyle behaviours, education and information needs to be better communicated to patients having elective surgery. Digital technologies (such as smartphone apps, tablets, activity trackers, and the internet) could support this. The aim of this review was to determine whether digital technologies are effective at supporting patients undergoing elective surgery to change their health behaviours, focusing on physical activity, weight, and dietary intake.

## Methods

This systematic review was conducted in accordance with PRISMA guidelines^25^ and registered with PROSPERO (CRD42019127972).

### Search strategy and study selection

A comprehensive and systematic literature search was conducted in March 2019 across six electronic databases including MEDLINE, Embase, CINAHL, PsycINFO, Web of Science, and Scopus. No limit on the publication date was applied. Experimental and observational studies that evaluated a digital intervention supporting behaviour change(s) in adult patients undergoing elective surgery (aged over 18 years), of any sex, ethnicity or nationality, during the preoperative or postoperative period, were included. The studies must have conducted an initial baseline measurement of participants and (at least) one follow-up measure, so as to evaluate whether a change in behaviour (physical activity levels, weight, and/or dietary habits) took place in the population group. Any study where the intervention focused on healthcare professionals, family and/or caregivers, or patients more than 2 years after surgery were excluded. Any studies that evaluated digital interventions from a psychological or quality-of-life point of view, or where the behaviour change related to disease screening (rather than active surgical care), were excluded. Qualitative studies, editorials, reviews, conference abstracts, and study protocols were also excluded. This review focused on elective surgical procedures, specifically bariatric, cancer and orthopaedic procedures, where patients require preoperative and postoperative lifestyle and health behaviour changes; abdominal, cardiac, gastrointestinal, gynaecological, and trauma operations were excluded.

Additional papers were identified via the grey literature within personal libraries of the authors, professional research networks, and by reference checking. Search terms are described in *Appendix S1*.

Titles and abstracts from the database search were reviewed by one author. Full texts were retrieved for articles that met the inclusion criteria for further evaluation, and for those that could not be rejected with certainty. Full-text articles were screened independently by two authors. Disagreements were resolved by discussion with a third reviewer where necessary.

### Data extraction and quality appraisal

Data extraction was carried out by two authors, using a customized data extraction form containing the following headings: study, intervention, population, behavioural change outcome, key findings, and study limitations. Quality and risk-of-bias assessment was conducted by two authors using the Joanna Briggs Institute critical appraisal tools^26^. This checklist includes questions relating to sampling, inclusion criteria, confounding, outcomes, and statistical analysis. All studies were assigned a methodological quality (bias) score for ease of reporting, expressed as a percentage. Interventions were grouped into three delivery time points for analysis: preoperative interventions (implemented before the surgical procedure); postoperative interventions (implemented after the surgical procedure); and preoperative and postoperative interventions (implemented before and continued after operation).

### Analysis and synthesis

A narrative synthesis describing studies thematically was undertaken. Studies reported heterogeneous measures so a meta-analysis was not possible. Overall effectiveness in supporting behavioural change in surgical patients was reported in terms of the delivery method, timing of intervention delivery, and theoretical underpinning of the digital interventions.

## Results

**Fig. 1:**
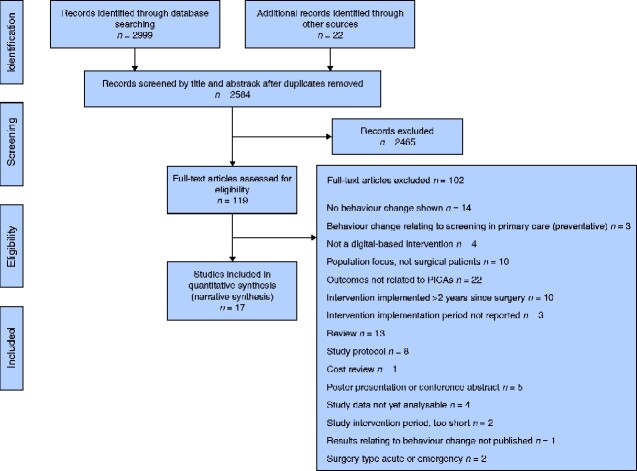
PRISMA flow chart showing selection of articles for review PICO, Population, Intervention, Comparison, Outcomes.

Initially 2999 citations were screened. An additional 22 studies were identified by hand-searching and grey literature search. After removal of duplicates and applying the eligibility criteria, 17 studies were included (*Fig. 1*). Ten of these were RCTs; the remaining seven included feasibility and efficacy studies, controlled observational studies, and a study employing a pre–post-test design.

These studies were published between 2011 and 2019. They were conducted in seven different countries, including the USA (5)^27–31^, the Netherlands (4)^32–35^, Canada (3)^15^^,^^36^^,^^37^, New Zealand (2)^38^^,^^39^, South Korea (1)^40^, Australia (1)^41^, and Spain (1)^42^. The studies included a total of 4923 surgical patients.

The studies covered three different surgery types: bariatric surgery (10 studies), cancer surgery (5), and orthopaedic surgery (2). Further study characteristics, including the timing and behaviours targeted for change, are detailed in *Table 1*. The studies varied in intervention delivery method, duration, and frequency of use. Two articles^35^^,^^42^ did not report any statistical analysis of the results. Of the remaining 15 papers, nine reported a significant effect indicating a change in health behaviours (*P* ≤ 0.050).

**Table 1:** Study characteristics

Reference	Type of surgery	Intervention target			Behaviour change target			Population size in intervention group	Participant sex	Control or comparator group
		Before surgery	After surgery	Before and after surgery	Physical activity	Weight	Diet			
Baillot *et al*.^36^	Bariatric	×			×			6	F	Yes
Bradley *et al*.^27^	Bariatric		×			×		20	F + M	No
Coleman *et al*.^28^	Bariatric		×		×			26	F + M	Yes
Doiron-Cadrin *et al*.^15^	Orthopaedic, TKA + THA	×			×			12	F + M	Yes
Kanera *et al*.^32^	Cancer, mixed		×		×		×	265	F + M	Yes
Kanera *et al*.^33^	Cancer, mixed		×		×		×	231	F + M	Yes
King *et al*.^29^	Bariatric			×	×			310	F + M	Yes
Lauti *et al*.^38^	Bariatric		×			×		47	F + M	Yes
Lee *et al*.^40^	Cancer, breast		×		×		×	29	n.r.	Yes
Lemanu *et al*.^39^	Bariatric	×			×			44	F + M	Yes
Mayer *et al*.^30^	Cancer, colon		×		×			144	F + M	Yes
Mundi *et al*.^31^	Bariatric	×			×	×		30	F + M	No
Ormel *et al*.^34^	Cancer, mixed			×	×			16	F + M	Yes
Padwal *et al*.^37^	Bariatric	×				×		225	F + M	Yes
Russell *et al*.^41^	Orthopaedic, TKA		×		×			31	F + M	Yes
Tenhagen *et al*.^35^	Bariatric			×		×		14	F + M	No
Vilallonga *et al*.^42^	Bariatric		×			×		10	F + M	Yes

TKA, total knee arthroscopy; THA, total hip arthroscopy; n.r., not reported.

### Study quality

The overall methodological quality of included studies was good, with a mean quality score of 69 per cent (*Appendix S2*). Scores ranged from 54 per cent^30^^,^^39^ to 100 per cent^34^^,^^36^.

### Delivery of intervention

Different digital technologies were used to deliver the interventions, including internet-based interventions (telemedicine, emails, and e-platforms)^15^^,^^32^^,^^33^^,^^36^^,^^37^^,^^40^^,^^41^, phone-based interventions (text messaging and apps)^30^^,^^34^^,^^38^^,^^39^, wearable interventions (activity monitors)^29^, and combination interventions (more than 1 form of digital technology to support health behaviour change)^27^^,^^28^^,^^31^^,^^35^^,^^42^. *Appendix S3* provides an overview of the method of delivery, target, and engagement rate of interventions.

### Internet-based interventions

Seven studies used internet-based interventions to promote health behaviour change, three^15^^,^^36^^,^^41^ of which employed telemedicine, and the remaining four^32^^,^^33^^,^^37^^,^^40^ used an e-platform system, made up of educational modules. None of the three telemedicine studies led to change in health behaviours, although the authors did recognize the potential benefits of using this method of delivery to overcome provision and geographical barriers^41^.

The e-platform approach produced health behaviour change across three of the four studies^32^^,^^33^^,^^40^. Two studies^32^^,^^33^ employed the Kanker Nazorg Wijzer e-platform to provide personalized educational modules concerning physical activity (in minutes of exercise per week) and diet (vegetable consumption in grams per day) to patients after cancer surgery. Kanera and colleagues^32^ reported how the intervention group improved moderate physical activity (by 150.73 min/week; *P* = 0.037) compared with control over a 6-month period, and this improvement was sustained over 12 months (*P* = 0.011). However, the increased vegetable consumption (grams per day; *P* = 0.027) over the 6-month period was not sustained at 12 months (*P* = 0.132). They also demonstrated that improvements in physical activity were significantly more successful in younger patients (aged less than 57 years) than older ones, over 6 months (minutes per week; *P*  = 0.04) and 12 months (minutes per week; *P* < 0.010)^33^. This echoes findings from previous work that showed how younger cancer survivors were more likely to improve their physical activity levels compared with older survivors, possibly owing to their perceptions of future risk^43^^,^^44^.

Another study^40^ focused on a web-based self-management exercise and diet intervention e-platform to support patients to improve exercise and dietary intake health behaviours after breast cancer surgery. The results demonstrated an improvement in diet (servings of fruit and vegetables per day; *P* = 0.001) and physical activity levels (minutes of exercise per week; *P* < 0.001) compared with the control.

### Phone-based interventions

Four studies delivered health behaviour change interventions using phone-based methods, two^38^^,^^39^ through text messaging services and two^30^^,^^34^ through smartphone apps. Lemanu and colleagues^39^ found that text message delivery over a 4–6-week period was successful in improving bariatric patient adherence to preoperative exercise (median days of exercise per week; *P* < 0.050), although this improvement was not sustained at 6 weeks’ postoperative follow-up. Ormel *et al.*^34^ showed significant improvements in physical activity in patients before and after cancer surgery with app use, which was not maintained at the 12-week follow-up. Mayer and co-workers^30^ also reported an improvement in physical activity in patients after surgery for colonic cancer with the SurvivorCHESS app. However, this improvement was no different from that in control patients (minutes of moderate and vigorous physical activity per week; *P* = 0.122) and only lasted as long as the intervention.

### Wearable interventions

King and colleagues^29^ provided participants with a wearable digital activity monitor (which tracked physical activity, including daily step counts and active minutes) to use alongside self-reporting physical activity levels in a paper diary, from 1 week before to 1 year after surgery. More participants changed from inactive to active, than from active to inactive, over the intervention period (minutes of exercise per week; *P* < 0.001). By using the diary, more participants self-reported physical activity levels improving from less than 150 min/week before surgery to 150 min/week or more at 1 year after operation (*P* < 0.001). The activity monitor recorded an increase in the number of steps per day and active minutes per day from before to 1 year after surgery (both *P* < 0.001).

### Combination interventions

Five studies used a combination of different digital approaches to motivate health behaviour change in patients undergoing bariatric surgery. One study^27^ used a combination of three digital elements (triple approach) and the other four^28^^,^^31^^,^^35^^,^^42^ used a dual approach. One study^31^ trialled a combination intervention before surgery and three studies^27^^,^^28^^,^^42^ after operation, and one study^35^ implemented the combined intervention both before and after operation across the surgical journey. Of the five combination interventions, three studies^27^^,^^28^^,^^31^ demonstrated behavioural change improvements, and two^35^^,^^42^ did not perform a statistical analysis.

In a triple-approach study, Bradley and co-workers^27^ implemented an e-platform in combination with an app and online log to investigate efficacy of reduced weight regain after bariatric surgery. Educational information was delivered through the e-platform and daily calorie intake calculated using an app. At completion of the intervention, 10 of 11 participants demonstrated weight loss or weight stabilization (in kilograms; *P* = 0.01). Weight loss was maintained at 3 months’ follow-up.

Coleman *et al.*^28^ implemented a dual approach, whereby participants used a form of wearable technology (pedometer) in combination with online activity logging to complement postoperative exercise programmes. An improvement was demonstrated in participants’ 6-min walk test (distance in metres; *P* = 0.001) during the intervention period and maintained at 6-month follow-up.

Mundi and colleagues^31^ employed a dual-approach intervention, consisting of an educational app and a daily text message service, for 12 weeks before bariatric surgery. At study completion there was a reduction in weight (in kilograms; *P* = 0.06) and BMI (kilograms per square metre; *P* < 0.001), and an increase in physical activity (minutes of vigorous activity per week; *P* = 0.04) in the intervention group.

Patients tracked their real-time weekly parameters before and after bariatric surgery, by using digital weighing scales (technology at home) connected to an online log in another dual-approach study^35^. On study completion, participant mean(s.d.) BMI decreased from 44.7(4.6) to 30.6(4.2) kg/m^2^; the mean estimated weight loss was 72(19.1) per cent and the mean BMI change was 32 per cent. Vilallonga and co-workers^42^ also employed a dual approach; WiFi-enabled weighing scales were used to log weight loss on an online account and members of the surgical team used e-mail to liaise with patients on postoperative weight-loss progress. The results demonstrated improvements in the mean percentage estimated weight loss, with the intervention group losing 65.3 per cent compared with 58.2 per cent for control. The mean postoperative BMI was 32.7 and 33.2 kg/m^2^ respectively.

### Timing of intervention delivery

*Appendix S3* shows details of the timing of each intervention in the studies, specifically how long patients used the interventions (intervention period) and their active engagement (retention rates). Four studies^15^^,^^31^^,^^36^^,^^37^ initiated interventions 12 weeks before surgery and one^39^ 4–6 weeks before operation. Nine studies used postoperative interventions, with some patients beginning almost immediately after surgery with a rehabilitation focus^41^, some during follow-up monitoring^27^^,^^32^^,^^33^^,^^40^^,^^42^, and up to 2 years after surgery in three studies^28^^,^^30^^,^^38^. The overall intervention period in the included studies differed substantially, the shortest being 6 weeks^41^ and the longest continuing over 12 months^40^. The preoperative and postoperative intervention by Ormel *et al.*^34^ was initiated following the decision to undergo surgery, and was continued for 12 weeks after operation. Tenhagen and co-workers^35^ also initiated the intervention after the surgical decision had been made, but continued for 12 months after the procedure, whereas King and colleagues^29^ initiated the intervention for 7 days in the week before surgery and repeated the intervention for another 7-day interval 1 year after operation.

Overall retention rates over the intervention period were high; only one study^27^ had a retention rate below 60 per cent. Four studies reported 100 per cent retention rates, including two with preoperative interventions^36^^,^^39^, one with a postoperative intervention^42^, and one with intervention before and after surgery^34^.

### Theoretical underpinning: behaviour change theories

Eight^27^^,^^28^^,^^30^^,^^32–34^^,^^38^^,^^40^ of the 17 studies referred to behaviour change theories or frameworks, either as a way of designing the intervention or for analysis of the results. Across these, social cognitive theory was used twice^32^^,^^33^, whereas theories such as acceptance and commitment therapy^27^, the trans-theoretical model^40^, self-determination theory^30^, the behaviour change wheel^38^, and goal-setting^28^ were used once. Ormel and colleagues^34^ did not specify which behavioural change theory informed the design of their app. Of the eight studies, six produced significant improvements in health behaviour changes (*P* ≤ 0.05) relating to reduced weight regain^27^, increased physical activity^28^^,^^34^, and improved lifestyle choices for physical activity and diet^32^^,^^33^^,^^40^.

## Discussion

In patients undergoing elective surgery, various forms of digital technology can support behaviour change successfully, in particular physical activity, dietary intake, and weight loss. The duration of behaviour change has proven to be variable, with some technologies demonstrating more success on a short-term basis. Three factors were identified that could contribute to digital technology effectiveness in the elective surgical population: delivery of an intervention, timing of the intervention, and behavioural change theories underpinning the intervention design.

High overall retention rates across studies indicate the acceptability of modern technologies in surgical care. This is not an unusual finding, with previous research supporting the success of digital technology overlap from social to health-related purposes^45–47^. High satisfaction rates among intervention group participants were seen in the internet-based studies, with 100 per cent reporting their overall satisfaction with the delivery format^15^, and 96 per cent attendance recorded for the telemedicine intervention group compared with 80 per cent for the control^36^. Padwal and colleagues^37^ concluded that e-platforms were often more expensive and labour-intensive to produce and run.

Although none of the studies using telemedicine demonstrated improvements in health behaviours, the authors acknowledged many benefits underpinning this delivery method. These included reduced travel to face-to-face appointments^32^^,^^33^, increased accessibility to healthcare services for those who are geographically, economically or functionally disadvantaged^36^, and improved continuity of care with the same physician working to programme completion^41^. This adds to the already growing body of literature supporting the wide-ranging opportunities that telemedicine interventions present^48^. Specifically, in a surgical context, this can reduce the need for in-person consultations before and after surgery^49^^,^^50^. The benefits of phone-based interventions included convenience for the patient (accessible at any time), low cost, and user-friendliness^30^^,^^38^^,^^39^. A higher level of sophistication, such as text messages that allow a response, offers more personalized advice as well as the possibility to link with self-monitoring applications to track progress, which may produce superior results^38^. Newer forms of delivery, such as wearable technologies, have increased in popularity over recent years, yet only two studies used wearable technologies in this review. One wearable was successful in isolation^29^ and one in combination^28^.

There were no interventions that included digitally based peer support networks in this review. Peer forums supporting and motivating preoperative and postoperative lifestyle changes have demonstrated success in past studies^51–53^. Peer support has been found to enhance the effectiveness of behaviour change, with authors postulating how this may increase motivation and adoption of social-norm approaches through social interactions^54–57^.

The optimum value of intervention timings, specifically initiation, duration, and frequency, on outcomes is unclear. Other factors such as the surgical procedure (or the underlying disease triggering surgery) may contribute to variation in behavioural change, and may in fact determine the timing of when, for how long, and how often patients engage with digital technologies. It would appear that preoperative digital interventions are beneficial in cementing a culture of behaviour change for the patient at the earliest opportunity, capitalizing on the surgical teachable moment^58–60^. The challenge is continuing the intervention after surgery in an attempt to sustain health behaviour change and obtain greater improvements in outcomes^61^.

This review is subject to some limitations. Study outcome measures were heterogeneous, often adapted to the specific population rather than for undergoing surgery in general. This made it difficult to judge the optimum approach(es) responsible for contributing to significant behaviour change in each cohort. Although it was possible to identify elements of intervention delivery and timing that may be effective for supporting surgical patients, the most important and effective element could not be determined. It was also unclear which combination(s) of intervention delivery approaches would be optimal. In a world where digital technologies develop at rapid pace, and are implemented more than ever within healthcare systems, these components should be established in order to have maximal effectiveness in supporting behaviour change in patients undergoing elective surgery, thus improving surgical quality and safety.

## Funding

Dr WE Harker PhD Studentship, Newcastle University.

## Supplementary Material

zraa009_Supplementary_Data
