# Supplementary material for: Digital technology to support lifestyle and health behaviour changes in surgical patients: systematic review
Source: BJS Open. 2020 Dec 28;5(2):zraa009. doi: 10.1093/bjsopen/zraa009 (PMC7944850; doi:10.1093/bjsopen/zraa009)
Supplement: zraa009_Supplementary_Data [file zraa009_supplementary_data.docx]

**Appendices:**

Appendix A: PRISMA 2009 checklist

Appendix B: Database search terms

Appendix C: Study quality

Appendix D: Method of delivery, target, and engagement rate of interventions

Appendix A: PRISMA 2009 checklist

| **Section/topic** | **#** | **Checklist item** | **Reported on page # of submitted version** |
| --- | --- | --- | --- |
| **TITLE** | | |  |
| Title | 1 | Identify the report as a systematic review, meta-analysis, or both. | i |
| **ABSTRACT** | | |  |
| Structured summary | 2 | Provide a structured summary including, as applicable: background; objectives; data sources; study eligibility criteria, participants, and interventions; study appraisal and synthesis methods; results; limitations; conclusions and implications of key findings; systematic review registration number. | 2 |
| **INTRODUCTION** | | |  |
| Rationale | 3 | Describe the rationale for the review in the context of what is already known. | 4 |
| Objectives | 4 | Provide an explicit statement of questions being addressed with reference to participants, interventions, comparisons, outcomes, and study design (PICOS). | 2-4 |
| **METHODS** | | |  |
| Protocol and registration | 5 | Indicate if a review protocol exists, if and where it can be accessed (e.g., Web address), and, if available, provide registration information including registration number. | 5 |
| Eligibility criteria | 6 | Specify study characteristics (e.g., PICOS, length of follow-up) and report characteristics (e.g., years considered, language, publication status) used as criteria for eligibility, giving rationale. | 5-6 |
| Information sources | 7 | Describe all information sources (e.g., databases with dates of coverage, contact with study authors to identify additional studies) in the search and date last searched. | 5 |
| Search | 8 | Present full electronic search strategy for at least one database, including any limits used, such that it could be repeated. | 6 + Table 1 in Supplementary File (Appendix C) |
| Study selection | 9 | State the process for selecting studies (i.e., screening, eligibility, included in systematic review, and, if applicable, included in the meta-analysis). | 7 |
| Data collection process | 10 | Describe method of data extraction from reports (e.g., piloted forms, independently, in duplicate) and any processes for obtaining and confirming data from investigators. | 7 |
| Data items | 11 | List and define all variables for which data were sought (e.g., PICOS, funding sources) and any assumptions and simplifications made. | 6-8 + Table 2 in Supplementary File (Appendix D) |
| Risk of bias in individual studies | 12 | Describe methods used for assessing risk of bias of individual studies (including specification of whether this was done at the study or outcome level), and how this information is to be used in any data synthesis. | 9 + Table 3 in Supplementary File (Appendix E) |
| Summary measures | 13 | State the principal summary measures (e.g., risk ratio, difference in means). | N/A |
| Synthesis of results | 14 | Describe the methods of handling data and combining results of studies, if done, including measures of consistency (e.g., I^2^) for each meta-analysis. | N/A |
| Risk of bias across studies | 15 | Specify any assessment of risk of bias that may affect the cumulative evidence (e.g., publication bias, selective reporting within studies). | N/A |
| Additional analyses | 16 | Describe methods of additional analyses (e.g., sensitivity or subgroup analyses, meta-regression), if done, indicating which were pre-specified. | N/A |
| **RESULTS** | | |  |
| Study selection | 17 | Give numbers of studies screened, assessed for eligibility, and included in the review, with reasons for exclusions at each stage, ideally with a flow diagram. | 8 + Figure 1 in Supplementary File (Appendix A) |
| Study characteristics | 18 | For each study, present characteristics for which data were extracted (e.g., study size, PICOS, follow-up period) and provide the citations. | 8-9 + Table 2 in Supplementary File (Appendix D) |
| Risk of bias within studies | 19 | Present data on risk of bias of each study and, if available, any outcome level assessment (see item 12). | 9 + Table 3 in Supplementary File (Appendix E) |
| Results of individual studies | 20 | For all outcomes considered (benefits or harms), present, for each study: (a) simple summary data for each intervention group (b) effect estimates and confidence intervals, ideally with a forest plot. | 32-36 + Table 2 in Supplementary File (Appendix D) |
| Synthesis of results | 21 | Present results of each meta-analysis done, including confidence intervals and measures of consistency. | N/A |
| Risk of bias across studies | 22 | Present results of any assessment of risk of bias across studies (see Item 15). | N/A |
| Additional analysis | 23 | Give results of additional analyses, if done (e.g., sensitivity or subgroup analyses, meta-regression [see Item 16]). | N/A |
| **DISCUSSION** | | |  |
| Summary of evidence | 24 | Summarize the main findings including the strength of evidence for each main outcome; consider their relevance to key groups (e.g., healthcare providers, users, and policy makers). | 19-24 |
| Limitations | 25 | Discuss limitations at study and outcome level (e.g., risk of bias), and at review-level (e.g., incomplete retrieval of identified research, reporting bias). | 24 |
| Conclusions | 26 | Provide a general interpretation of the results in the context of other evidence, and implications for future research. | 24-25 |
| **FUNDING** | | |  |
| Funding | 27 | Describe sources of funding for the systematic review and other support (e.g., supply of data); role of funders for the systematic review. | 25 |

*From:*  Moher D, Liberati A, Tetzlaff J, Altman DG, The PRISMA Group (2009). Preferred Reporting Items for Systematic Reviews and Meta-Analyses: The PRISMA Statement. PLoS Med 6(6): e1000097. doi:10.1371/journal.pmed1000097

For more information, visit: **www.prisma-statement.org**.

Appendix B: Database search terms

Full search strategy for all journals = [all terms in column 1 linked using ‘OR’] AND [all terms in column 2 linked using ‘OR’] AND [all terms in column 4 for each surgery type linked with ‘OR’]

OVID Platforms Search Strategy

| **1. Digital Tech Intervention** | **2. Sustained** | **3. Behaviour Change** | **4. Surgery requiring a Health Behaviour Change** | | |
| --- | --- | --- | --- | --- | --- |
|  |  |  | **Cancer Surgery** | **Bariatric Surgery** | **Orthopaedic Surgery** |
| digital technology.mp. | Identified using inclusion/exclusion criteria after searching | exp Health Behavior/ | cancer.mp. | exp bariatrics/ | orthopaedic.mp. |
| digital intervention.mp. |  | health behavior.mp. | cancer patient.mp. | bariatrics.mp. | knee replacement.mp. |
| eHealth.mp. |  | exp Life Style/ | exp cancer survivors/ | exp bariatric surgery/ | hip replacement.mp. |
| mHealth.mp. |  | life style.mp. | cancer survivor.mp. | bariatric surgery.mp. | arthroplasty.mp. |
| exp TELEMEDICINE/ |  | exp Healthy Lifestyle/ | cancer surgery.mp. | weight loss surgery.mp. | joint.mp. |
| telemedicine.mp. |  | healthy lifestyle.mp. |  | exp obesity management/ | joint surgery.mp. |
| telehealth.mp. |  | Lifestyle change.mp. |  | obesity management.mp. | exp general surgery/ |
| digital healthcare.mp. |  | exp Health Promotion/ |  | exp overweight/ | exp elective surgical procedures/ |
| smartphone application.mp. |  | health promotion.mp. |  |  | surgery.mp. |
| exp SMARTPHONE/ |  | behavio* change.mp. |  |  | general surgery.mp. |
| smartphone.mp. |  | health information.mp. |  |  | elective surgery.mp. |
| exp Cell Phone/ |  | exp Health Education/ |  |  | exp preoperative care/ |
| cell phone.mp. |  | health education.mp. |  |  | exp postoperative care/ |
| exp Mobile Applications/ |  | exp Health Risk Behaviors/ |  |  | exp perioperative care/ |
| mobile applications.mp. |  | health risk behaviors.mp. |  |  | preoperative care.mp. |
| exp Internet/ |  | exp Attitude to Health/ |  |  | postoperative care.mp. |
| internet.mp. |  | attitude to health.mp. |  |  | perioperative care.mp. |
| web-based.mp. |  | social cognitive theory.mp. |  |  | surgical pathway.mp. |
| internet-based.mp. |  | SCT.mp. |  |  |  |
| computer-based.mp. |  | exp Self Efficacy/ |  |  |  |
| exp Computer-Assisted Instruction/ |  | self efficacy.mp. |  |  |  |
| exp Wearable Electronic Devices/ |  | transtheoretical model of change.mp. |  |  |  |
| wearable technology.mp. |  | transtheoretical model.mp. |  |  |  |
| exp Fitness trackers/ |  | stages of change.mp. |  |  |  |
| activity tracker.mp. |  |  |  |  |  |

PsycINFO Search Strategy

| **Digital Tech Intervention** | **Sustained** | **Behaviour Change** | **Surgery requiring a Health Behaviour Change** | | |
| --- | --- | --- | --- | --- | --- |
|  |  |  | **Cancer Surgery** | **Bariatric Surgery** | **General Surgery** |
| digital technology.mp. | Identified using inclusion/exclusion criteria after searching | exp Health Behavior/ | cancer.mp. | bariatrics.mp. | general surgery.mp. |
| digital intervention.mp. |  | health behavior.mp. | cancer patient.mp. | exp bariatric surgery/ | elective surgery.mp. |
| eHealth.mp. |  | life style.mp. | cancer survivor.mp. | bariatric surgery.mp. | surgery.mp. |
| mHealth.mp. |  | healthy lifestyle.mp. | cancer surgery.mp. | weight loss surgery.mp. | preoperative care.mp. |
| exp TELEMEDICINE/ |  | Lifestyle change.mp. |  | exp obesity management/ | postoperative care.mp. |
| telemedicine.mp. |  | exp Health Promotion/ |  | obesity management.mp. | perioperative care.mp. |
| telehealth.mp. |  | health promotion.mp. |  | obesity surgery.mp. | surgical pathway.mp. |
| digital healthcare.mp. |  | behavio* change.mp. |  | exp weight control/ | surgical recovery.mp. |
| smartphone application.mp. |  | health information.mp. |  | weight control.mp. | preparation for surgery.mp. |
| smartphone.mp. |  | exp Health Education/ |  | exp overweight/ | exp surgical patients/ |
| cell phone.mp. |  | health education.mp. |  | overweight.mp. |  |
| exp Internet/ |  | health risk behaviors.mp. |  |  |  |
| internet.mp. |  | attitude to health.mp. |  |  |  |
| web-based.mp. |  |  |  |  |  |
| internet-based.mp. |  |  |  |  |  |
| computer-based.mp. |  |  |  |  |  |
| exp Computer-Assisted Instruction/ |  |  |  |  |  |

Web of Science Search Strings

| **Digital Tech Intervention** | **Sustained** | **Behaviour Change** | **Surgery requiring a Health Behaviour Change** | | |
| --- | --- | --- | --- | --- | --- |
|  |  |  | **Cancer Surgery** | **Bariatric Surgery** | **General Surgery** |
| TS=(digital technology OR digital intervention OR eHealth OR mHealth OR telemedicine OR telehealth OR digital healthcare OR smartphone application OR smartphone OR cell phone OR internet OR web-based OR internet-based OR computer-based)) *AND* **LANGUAGE:** (English) | Identified using inclusion/exclusion criteria after searching | TS=(health behavio* OR health behavio* change OR healthy lifestyle OR lifestyle change OR health promotion OR behavio* change OR health education OR health risk behaviors)) *AND* **LANGUAGE:** (English) | TS=(cancer OR cancer patient OR cancer survivor OR cancer pathway)  AND  TS=(cancer surgery)) | TS=(bariatrics OR bariatric surgery OR weight loss surgery OR obesity management OR obesity surgery OR weight loss management OR weight control OR overweight) | TS=(surgery OR general surgery OR elective surgery OR preoperative care OR postoperative care OR perioperative care OR surgery pathway OR preparation for surgery) |

Appendix C: Study quality

Study quality

| **Author, year** | **Study design** | **Critical bias score** | | | | | | | | | | | | | **Total Score** | |
| --- | --- | --- | --- | --- | --- | --- | --- | --- | --- | --- | --- | --- | --- | --- | --- | --- |
|  |  | 1 | 2 | 3 | 4 | 5 | 6 | 7 | 8 | 9 | 10 | 11 | 12 | 13 | (n) | (%) |
| Baillot et al., 2017[36] | Pre-/post- test design | Y | Y | Y | Y | Y | Y | Y | Y | Y |  |  |  |  | 9/9 | 100 |
| Bradley et al., 2017[27] | Feasibility and efficacy | Y | X | X | N | Y | Y | X | Y | Y |  |  |  |  | 5/9 | 56 |
| Coleman et al., 2017[28] | RCT | Y | ? | N | N | ? | ? | Y | Y | Y | Y | Y | Y | Y | 8/13 | 62 |
| Doiron-Cadrin et al., 2019[15] | RCT | Y | Y | Y | X | ? | ? | Y | Y | Y | Y | ? | Y | Y | 9/13 | 69 |
| Kanera et al., 2016[32] | RCT | Y | Y | N | N | ? | ? | Y | Y | Y | Y | Y | ? | Y | 8/13 | 62 |
| Kanera et al., 2017[33] | RCT | Y | Y | N | X | ? | ? | Y | Y | Y | Y | Y | ? | Y | 8/13 | 62 |
| King et al., 2012[29] | Observational | Y | N | Y | Y | Y | ? | Y | N | Y |  |  |  |  | 6/9 | 67 |
| Lauti et al., 2018[38] | RCT | Y | Y | Y | N | X | Y | Y | Y | Y | Y | ? | ? | Y | 9/13 | 69 |
| Lee et al., 2014[40] | RCT | Y | Y | N | Y | X | X | Y | Y | Y | Y | ? | Y | Y | 8/13 | 62 |
| Lemanu et al., 2018[39] | RCT | N | N | Y | N | X | Y | Y | Y | Y | Y | Y | N | X | 7/13 | 54 |
| Mayer et al., 2018[30] | RCT | ? | ? | Y | X | N | N | Y | N | Y | Y | Y | Y | Y | 7/13 | 54 |
| Mundi et al., 2015[31] | Feasibility | Y | Y | X | N | Y | Y | X | Y | Y |  |  |  |  | 6/9 | 67 |
| Ormel et al., 2018[34] | Feasibility | Y | Y | Y | Y | Y | Y | Y | Y | Y |  |  |  |  | 9/9 | 100 |
| Padwal et al., 2017[37] | RCT | Y | Y | N | X | Y | Y | Y | Y | Y | Y | Y | Y | Y | 11/13 | 85 |
| Russell et al., 2011[41] | RCT | Y | Y | N | X | Y | Y | Y | Y | Y | Y | ? | Y | Y | 10/13 | 77 |
| Tenhagen et al., 2016[35] | Feasibility and efficacy | Y | X | X | N | Y | Y | X | Y | Y |  |  |  |  | 5/9 | 56 |
| Vilallonga et al., 2013[42] | Observational | Y | N | Y | Y | Y | Y | Y | N | N |  |  |  |  | 6/9 | 67 |
|  | | | | | | | | | | | | | | | **Average** | **69** |
| **Key:** Y = yes, N = no, X = not applicable, ? = unclear, RCT = randomised controlled trial | | | | | | | | | | | | | | | | |

Appendix D: Method of delivery, target, and engagement rate of interventions

Table 2: Method of delivery, target, and engagement rate of interventions

| **Author, year** | **Method of intervention delivery** | **Delivery platform** | **Intervention description** | **Intervention target** | **Intervention period** | **Statistically significant HBC seen (p≤0.05) (Y/N)** | **Retention rate (%)** |
| --- | --- | --- | --- | --- | --- | --- | --- |
| Baillot et al., 2017^(36)^ | Internet-based | TM | Telemedicine intervention: “TelePreSET” in-house strength and endurance exercise training via videoconferencing. | Pre-op | 12 weeks | N | 100 |
| Bradley et al., 2017^(27)^ | Combination | SA, eP, OL | Mixed-delivery digital interventions:  - Module-based intervention, delivered via an online eLearning platform.  - MyFitnessPal® smartphone app to document daily food diary.  - Online spreadsheet to document daily weight and calorie intake. | Post-op | 10 weeks | Y | 55 |
| Coleman et al., 2017^(28)^ | Combination | OL, WT | Mixed-delivery intervention: wearable technology and web-based activity logging, complementing group exercise sessions.  - 2 x weekly group exercise sessions,  - 3 days/week self-directed exercise,  - daily pedometer wear for real-time recording,  - daily logging of physical activity and steps via 10,000 steps website,  - and weekly counselling sessions via telephone. | Post-op | 6 months | Y | 81 |
| Doiron-Cadrin et al., 2019^(15)^ | Internet-based | TM | Telemedicine-based intervention: one-to-one tele-prehabilitation sessions (2x/week with a physical therapist) via tele-communication software (iPad), alongside PA log book to self-report exercise at home. | Pre-op | 12 weeks | N | 97 |
| Kanera et al., 2016^(32)^ | Internet-based | eP | Internet-based intervention: via an online portal (KNW), providing advice and support to patients, with personalised modules for education, including PA, diet, and QoL. | Post-op | 12 weeks | Y | 87 |
| Kanera et al., 2017^(33)^ | Internet-based | eP | Web-based intervention: computer tailored intervention via an online portal (KNW), providing advice and support to patients, with personalised modules for education, including PA, diet, and QoL. | Post-op | 6 months | Y | 83 |
| King et al., 2012^(29)^ | Wearable | WT | Wearable-based intervention: activity monitor provided by researchers to wear for the intervention period and PA diary to document self-reported activity levels for intervention period. | P&P | 1 week | Y | NR |
| Lauti et al., 2018^(38)^ | Phone-based | TX | Text message-based intervention: daily, one-way, text messages sent each to participants every morning for a 12 month period. | Post-op | 12 months | N | 90 |
| Lee et al., 2014^(40)^ | Internet-based | eP | Internet-based intervention: web-based self-management exercise and dietary intervention (WSEDI) aimed at enhancing PA and dietary behaviours through information, educational modules, and assessment modules, underpinned by behavioural change theories including action planning and goal setting. | Post-op | 12 weeks | Y | 98 |
| Lemanu et al., 2018^(39)^ | Phone-based | TX | Text message-based intervention: daily, one-way, text messages sent for 4-6 weeks prior to surgery to encourage/remind patients to keep exercising pre-operatively. | Pre-op | 4-6 weeks | Y | 100 |
| Mayer et al., 2018^(30)^ | Phone-based | SA | Smartphone-based app (Survivor CHESS): with components for PA tracking, peer-peer social networking, PA educational information, care planning, and one-to-one motivational messaging with coach (also provided with leaflets). | Post-op | 6 months | N | 82 |
| Mundi et al., 2015^(31)^ | Combination | SA, TX | Mixed-delivery: smartphone-delivered intervention, including: smartphone app consisting of educational modules (n=9) with assessments on completion (70% pass mark - modules were either nutrition related or physical activity related) and daily text messages encompassing lifestyle domains (including PA and meal planning). | Pre-op | 12 weeks | Y | 67 |
| Ormel et al., 2018^(34)^ | Phone-based | SA | Smartphone-based intervention: using a smartphone app (RunKeeper®) to track PA levels during and after cancer treatment in comparison with usual care. | P&P | 12 weeks | Y | 100 |
| Padwal et al., 2017^(37)^ | Internet-based | eP | Internet-based intervention: online modular intervention, delivered via an online eLearning platform, accessible any time over a three month period. | Pre-op | 12 weeks | N | 71 |
| Russell et al., 2011^(41)^ | Internet-based | TM | Internet-based intervention: weekly tele-rehabilitation exercise program to aid patient recovery following total knee arthroplasty (TKA). Once weekly, supervised, 45 minute session with clinician, encouraged to perform twice daily exercise. | Post-op | 6 weeks | N | 97 |
| Tenhagen et al., 2016^(35)^ | Combination | OL, TaH | Mixed-delivery: online-based monitoring of weight, via digital-internet connected scales. Online platform/dashboard for monitoring participant weights (baseline, current), graphical representations of progress, and digital-internet connected scales provided for weekly home measurements (weight data is transferred wirelessly and sent to central online database). | P&P | 12 months | SNP | 79 |
| Vilallonga et al., 2013^(42)^ | Combination | Email, TaH | Mixed-delivery intervention: TaH and email-based intervention: Remote virtual follow up assessments, email contact with surgeons, and online account with data to track weight loss progress. WiFi-enabled weighing scales to generate readings for BMI and percentage of fat and muscle tissue. All of the data stored electronically on their account, shared with surgeons, to track weight loss progress. Follow up assessments done via email, using shared real-time data. Data can be shared with social media if patient wanted. | Post-op | 3 months | SNP | 100 |
| **Key:** Y = yes, N = no, TM = telemedicine, SA = smartphone app, eP = e-platform, OL = online log, WT = wearable technology, TX = text message, TaH = technology at home (*e.g.* digital scales), QoL = quality of life, Pre-op = pre-operative target, Post-op = post-operative target, P&P = pre- and post-operative target, HBC = health behaviour change, SNP = statistical analysis not performed by authors. | | | | | | | |
